# Soybean Lecithin High in Free Fatty Acids for Broiler Chicken Diets: Impact on Performance, Fatty Acid Digestibility and Saturation Degree of Adipose Tissue

**DOI:** 10.3390/ani9100802

**Published:** 2019-10-14

**Authors:** Alberto Viñado, Lorena Castillejos, Ana Cristina Barroeta

**Affiliations:** Animal Nutrition and Welfare Service, Department of Animal and Food Science, Facultat de Veterinària, Universitat Autònoma de Barcelona, Bellaterra, 08193 Barcelona, Spain; Alberto.Vinado@uab.cat (A.V.); Ana.Barroeta@uab.cat (A.C.B.)

**Keywords:** broiler chickens, alternative energy source, soybean lecithin, phospholipids, vegetable acid oil, digestibility balance, free fatty acids, triacylglycerols

## Abstract

**Simple Summary:**

The search of alternatives for soybean oil, as a dietary energy source, has generated a lot of interest in broiler feeding due to economic and supply reasons. Soybean lecithin, as a co-product derived from the soybean oil degumming process, and its blending with other by-products derived from the vegetable oil refining process such as acid oils, may represent an alternative energy source for broiler chicken diets formulation. The current study has demonstrated that soybean lecithin high in free fatty acids can be included in grower–finisher diets, as a partial replacer of soybean oil or in combination with an acid oil, without impairing performance or fatty acid digestibility and causing minor changes in the fatty acid composition of the abdominal fat pad.

**Abstract:**

Two experiments were conducted to evaluate the inclusion of soybean lecithin with a high free fatty acid content (L) in starter and grower–finisher broiler diets, as well as its influence on performance, energy and fatty acid (FA) utilization and the FA profile of the abdominal fat pad (AFP). A basal diet was supplemented with soybean oil (S; Experiment 1) or acid oil (AO; Experiment 2) at 3%, and increasing amounts of L (1%, 2% and 3%) were included in replacement. The inclusion of L did not modify performance parameters (*p* > 0.05). The S replacement by L reduced energy and total FA utilization (*p* ≤ 0.05) in starter diets; however, in grower–finisher diets, a replacement up to 2% did not modify energy and FA utilization (*p* > 0.05). The AO substitution by L produced no modifications on energy and FA utilization (*p* > 0.05) during the starter phase, while the blend of 1% of AO and 2% of L resulted in the best combination in terms of the FA digestibility. The FA profile of the AFP reflected the FA composition of diets. The addition of L could replace, up to 2% or be blended with AO in broiler grower–finisher diets as an energy source.

## 1. Introduction

Co-products and by-products derived from the vegetable oil refining process may represent an interesting and economic alternative to conventional fat sources used in broiler feeding, such as soybean oil. During degumming, most phospholipids (PL) present in crude soybean oil are extracted, generating a co-product known as crude soybean lecithin. Lecithins are defined as a lipid mixture highly composed of PL, but they are also rich in glycolipids, carbohydrates and neutral lipids, such as triacylglycerols [1]. Soybean lecithin is an available low-cost energetic source [2] with a similar fatty acid (FA) profile to soybean oil [3,4]. In addition, its elevated surface-active PL content of soybean lecithin represents an added value as an emulsifier; hence, its dietary inclusion may improve fat absorption [5,6]. However, soybean lecithin has a high viscosity that hampers its inclusion during feed manufacturing. For this reason, in order to facilitate its homogeneous blending in feed, mixing lecithin at different ratios with acid or crude oils is a common practice [7]. On the other hand, vegetable acid oils derived from the chemical refining process of crude oils are normally composed of a large quantity of free fatty acids (FFA; 40%–60%) and represent an important source of energy [8,9]. Nevertheless, it has been observed that a high dietary FFA concentration may reduce energy utilization by impairing dietary fat solubilization in the gastrointestinal tract [5].

We hypothesized that soybean lecithin could be considered as an alternative energy source for broiler chicken diets, in replacement or combined with other fats, with no negative effects on the performance, nutrient digestibility, and FA composition of adipose tissue. Therefore, a total of two experiments were conducted to assess the potential use of a soybean lecithin high in FFA (L) as an alternative energy source in broiler feeding when combined with soybean oil (S; Experiment 1) or a monounsaturated vegetable acid oil (AO; Experiment 2). The evaluation was based on the study of the influence of L inclusion on performance, feed energetic content, FA digestibility, and, thus, the effect on the FA profile of the abdominal fat pad (AFP) of the broiler carcass.

## 2. Materials and Methods

### 2.1. Experimental Design and Diets

The experiments were performed at Servei de Granges i Camps Experimentals (Universitat Autònoma de Barcelona, Bellaterra, Spain), were in accordance with the European Union Guidelines (2010/63/EU), and were approved by the Animal Ethics Committee (CEEAH) of the same institution (number code: 4006). Two different trials of 38 days (d) each were performed with a feeding program in two phases: Starter (from 0 to 21 d) and grower–finisher (from 22 to 38 d). Experimental diets (Table 1) were based on wheat and soybean meal, presented in mash form, and were formulated to meet or exceed FEDNA (Fundación Española para el Desarrollo de la Nutrición Animal) requirements [10]. Furthermore, titanium dioxide (TiO_2_) was used as an inert marker at 5 g/kg in order to perform digestibility balances.

Experiment 1: A total of 96 Ross 308 newly hatched female broiler chickens were randomly assigned to one of four experimental treatments (six replicates/treatment) and allocated in cages (four birds/cage). A control basal diet was supplemented with S at 3% (S3), and increasing amounts of L (soybean lecithin blended with soybean acid oil in a 5:1 proportion) were included in replacement of S as added fat: 1% (S2–L1), 2% (S1–L2) and 3% (L3).

Experiment 2: A total of 120 Ross 308 newly hatched female broiler chickens were randomly assigned to one of five experimental treatments (six replicates/treatment) and allocated in cages (four birds/cage). A control basal diet was supplemented with AO (a 1:1 blend of olive pomace acid oil and sunflower acid oil) at 3% (AO3), and increasing amounts of L were included in replacement of AO: 1% (AO2–L1), 2% (AO1–L2) and 3% (L3). The S3 diet was included as a reference treatment.

### 2.2. Animal Husbandry and Controls

The animals were obtained from a local hatchery (Pondex S.A.U., Juneda, Spain), weighed, wing-tagged and randomly distributed in cages with a grid floor and a tray for excreta collection. The temperature and light program used was consistent with the specifications in the Ross 308 lineage management handbook [11], and the animals were allowed to consume feed and water ad libitum. Broiler body weight (BW) was recorded individually at 21 and 38 d post-hatch, whereas feed intake was measured by cage at 21 and 38 d post-hatch. The data were used to calculate the average daily gain (ADG), average daily feed intake (ADFI) and feed conversion ratio (FCR) from both phases and the overall results of each experiment. Mortality was recorded daily to adjust ADG and ADFI. Two nutritional balances were performed for each experiment between d 9 and 11 (starter period) and d 36 and 37 (grower–finisher period), where excreta samples (free of contaminants) were taken on each day of the digestibility balance (once per d), homogenized, freeze-dried, ground, and kept at 4 °C until further analysis. At the end of each experiment, all the animals used in both experiments were slaughtered in a commercial abattoir, and carcasses were recovered.

Carcasses (total BW excluding blood and feathers) were weighed, and the AFP (from the proventriculus surrounding the gizzard down to the cloaca) of each bird was removed and weighed in order to calculate the AFP carcass percentage. Furthermore, representative sample of the AFP of each bird was taken, pooled by replicate, and frozen at −20 °C for further analysis.

### 2.3. Laboratory Analyses

Experimental oil samples (S, L and AO) were chemically characterized, as shown in Table 2.

The FA composition was analyzed by gas chromatography following the methodology described by Guardiola et al. [12]. The acid value was determined according to International Organization for Standardization (ISO) 660 [13], and the acidity was expressed as the FFA percentage of oleic acid. In the case of the soybean lecithin high in FFA, the acetone insoluble matter was analyzed using the Ja 4–46 method from the American Oil Chemists’ Society (AOCS) [14], and the PL composition was determined by HPLC (D450 MT1, Kontron; Eching, Germany) according to the method described by Helmerich and Koehler [15].

Regarding the experimental feed samples, the proximate analysis was performed following AOAC methodology [16]: Ether extract (Method 920.39), crude protein (Method 968.06), ash (Method 942.05), dry matter (Method 934.01), and crude fiber (Method 962.09). The gross energy content was determined for oil, feed and excreta samples by an adiabatic bomb calorimeter (IKA-Kalorimeter system C4000; Staufen, Germany). Titanium dioxide was determined by inductively coupled plasma-optical emission spectrometry (ICP-OES; Optima 3200 RL, Perkin Elmer; Waltham, MA, USA) in Experiment 1, while it was determined in Experiment 2 by the method described by Short et al. [17].

The FA profile of the feed and excreta was analyzed by adding nonadecanoic acid (C19:0, Sigma-Aldrich Chemical Co.; St. Louis, MO) as an internal standard and following the method described by Sukhija and Palmquist [18], whereas in the case of the AFP, the method described by Carrapiso et al. [19] was used. The final extract obtained was injected into a gas chromatograph (HP6890, Agilent Technologies; Waldbronn, Germany) following the method conditions described by Cortinas et al. [20].

### 2.4. Calculations and Statistical Analysis

The apparent digestibility of FA (%) was calculated using the following equation:

The apparent digestibility of FA = 1 − {(TiO_2_ in feed/FA concentration in feed)/(TiO_2_ concentration in excreta/FA concentration in excreta)}.

The apparent metabolizable energy (AME) of the diets was calculated multiplying the apparent absorption of the gross energy by its corresponding diet gross energy. Both calculation formulas were in accordance with Rodriguez-Sanchez et al. [21].

Cage means were used as the experimental unit (six replicates/treatment) in performance (except BW), FA digestibility, the FA profile of the AFP, and the AME values of the diets. A Shapiro–Wilk test indicated a normal distribution of the data. In Experiment 1, data were analyzed by a one-way ANOVA using R Statistics (Version 3.3.1; R Core Team, Vienna, Austria), with treatment as the main factor. In Experiment 2, soybean oil treatment (S3) was compared against the AO3 treatment separately with a one-way ANOVA (S3 vs. AO3), whereas diets containing co-products and by-products were compared with a one-way ANOVA (AO3 vs. AO2–L1 vs. AO1–L2 vs. L3). Tukey’s multiple-range test was performed to determine whether means were significantly different (*p* ≤ 0.05). The linear model used was: Y_ij_ = µ + α_i_ + ε_j_, where *µ* is the global mean, *α* is the treatment effect, and ε is the residual error.

## 3. Results

### 3.1. Experimental Fats and Diets Composition

The FA profiles of S and L (Table 2) were similar regarding polyunsaturated FA (PUFA) content; nevertheless, L presented a higher content in saturated FA (SFA) and a lower content in monounsaturated FA (MUFA) than S. In the case of AO, oleic acid was the most abundant FA, followed by linoleic acid. Furthermore, the three added fats differed in their average unsaturated-to-saturated FA ratio (UFA:SFA), where S and AO presented higher average values (5.14 and 5.60, respectively) than L (3.74); the three fats also differed in their average polyunsaturated-to-saturated FA ratio (PUFA:SFA), where AO presented the lowest value (2.04), followed by L (2.82) and S (3.67). Concerning FFA content, AO presented the highest value, representing its main lipid molecular structure (52.9%), whereas L showed a medium average content (24.1%), and S showed the average lowest value (1.95%). Additionally, both S and AO presented higher average values of gross energy (39.8 and 39.5 MJ/kg, respectively) than L (34.4 MJ/kg).

The proximate analysis results and the FA profile of the experimental diets are shown in Table 3 (Experiment 1) and Table 4 (Experiment 2). The experimental treatments showed a similar macronutrient content, and their main differences were related to the FA profile and the energetic content. In Experiment 1, the replacement of S by L increased dietary SFA in starter (9.4%) and grower–finisher diets (11.9%), whereas a decrease in MUFA was observed (11.3% and 7.1% for starter and grower–finisher diets, respectively), causing a reduction in dietary the UFA:SFA. In Experiment 2, the replacement of AO by L increased dietary SFA (9.0% and 7.2%, for starter and grower–finisher diets, respectively) and dietary PUFA (28.4% and 36.8% for starter and grower–finisher diets, respectively). On the contrary, this replacement reduced the MUFA content (42.8% and 36.7% for starter and grower–finisher diets, respectively). The replacement of AO by L reduced the UFA:SFA, whereas it increased the PUFA:SFA. 

### 3.2. Growth Performance and Abdominal Fat Deposition

The growth performance and abdominal fat deposition parameters of Experiments 1 and 2 are shown in Table 5 and Table 6, respectively.

In both experiments, performance parameters were not affected by the replacement of the added fats (S and AO) by L in any phase, nor were the overall parameters of the experiments (*p* > 0.05). Nevertheless, in Experiment 2, the S replacement by AO impaired the feed conversion ratio in the grower–finisher phase and the global period of the experiment (*p* ≤ 0.05); the AO replacement by L tended to improve the feed conversion ratio (*p* = 0.055). Concerning the effect of dietary added fats on abdominal fat deposition, no significant differences were observed between experimental treatments (*p* > 0.05). 

### 3.3. Digestibility Balances

The influence of the added fats on the dietary feed AME and the FA digestibility in both feeding periods can be seen in Table 7 (Experiment 1) and Table 8 (Experiment 2).

The digestibility balance of Experiment 1 indicated, in starter diets, that the partial and total replacement of S by L (S2–L1, S1–L2, and L3) negatively affected the feed AME value (*p* < 0.001) and the FA digestibility. Animals fed diets with 2% and 3% of L (S1–L2 and L3) showed a lower total fatty acid (TFA; *p* = 0.017), MUFA (*p* = 0.026) and PUFA (*p* = 0.004) digestibility, and they tended to absorb SFA worse than animals fed S3 (*p* = 0.055). In the case of grower–finisher diets, animals fed L3 presented a lower feed AME (*p* < 0.001), and a lower TFA (*p* = 0.020), oleic acid (*p* < 0.001) and PUFA (*p* = 0.003) digestibility as compared to animals fed S3. However, no differences were observed between S3 and treatments with partial replacement by L (S2–L1 and S1–L2). 

Results from Experiment 2 showed that the S3 treatment presented a higher dietary AME and TFA digestibility than AO3 in both periods (*p* ≤ 0.05). Regarding the use of co-products (AO and L) as added fats, in the starter period, replacing AO by L led to no observable differences in the feed AME and the digestibility of TFA, SFA and MUFA (*p* > 0.05). Nevertheless, L3 presented a higher digestibility of linolenic acid (*p* = 0.011) in contrast to AO3. On the other hand, grower–finisher diets showed differences between treatments in the SFA, MUFA and PUFA digestibility. The total replacement of AO by L (L3) did not modify the dietary AME or the digestibility of TFA and SFA, but it caused a lower MUFA (*p* < 0.001) and a higher linolenic acid (*p* = 0.006) digestibility. The lowest feed AME value was observed in AO2–L1 (*p* < 0.001), which was consistent with the FA digestibility. The AO2–L1 treatment presented a lower TFA and MUFA digestibility than AO3 (*p* ≤ 0.05), and it presented a lower SFA digestibility than AO1–L2 and L3 (*p* < 0.01). Nonetheless, animals fed AO1–L2 did not show differences with the AO3 treatment and presented a higher MUFA digestibility in comparison to L3 (*p* < 0.001). 

### 3.4. Fatty Acid Composition of Abdominal Fat Adipose Tissue

The effect of dietary added fat on the FA composition of the AFP can be seen in Table 9. Total replacement of S by L increased SFA, in particular palmitic acid concentration (*p* < 0.01), whereas it reduced the UFA:SFA and the PUFA:SFA (*p* < 0.01). Furthermore, a tendency for a reduction of linoleic acid concentration (*p* = 0.069) was observed. In contrast to S3, animals feed AO3 presented an AFP with a higher MUFA concentration, concretely oleic acid (*p* ≤ 0.05), and a lower PUFA content, concretely linoleic and linolenic acid (*p* ≤ 0.05), thus reducing the PUFA:SFA (*p* ≤ 0.05). Finally, the use of L as a substitute for AO caused an increase in PUFA, specifically linoleic and linolenic acid (*p* < 0.01), and a reduction in the MUFA content (*p* < 0.01). In this case, the PUFA:SFA increased as long as L replaced AO (*p* = 0.014).

## 4. Discussion

### 4.1. Chemical Composition of the Experimental Fats and Diets

The gross energy content of the added fats indicated that L resulted in being less energetic than S and AO. This fact is a direct consequence of PL releasing less energy than triacylglycerol and FFA. Furthermore, the L included in both experiments contained high levels of FFA (24.1%) because it was blended with soybean acid oil. The standard FFA content of soybean lecithin products is normally established at between 1.0% and 3.0% [1,22]. It is important to mention that available literature regarding the use of a soybean lecithin high in FFA in monogastric nutrition is scarce, and the literature review was based on studies that used a regular soybean lecithin with a lower FFA content. The chemical composition of the experimental diets reflected the FA profile of the added fats. The dietary UFA:SFA was reduced, as L was included in the replacement of S, which was also reported by Soares and Lopez-Bote [3].

### 4.2. Growth Performance and Abdominal Fat Deposition

The inclusion of L as a substitute for S did not lead to any negative effect on growth efficiency. Results agree with Azman and Cifti [23], who observed that a partial replacement (50%) of soybean oil by a soybean lecithin (4% and 6% of total added fats for starter and grower–finisher diets, respectively) did not modify final the BW or the global feed conversion ratio. However, the replacement of S by AO reduced feed conversion efficiency in the grower–finisher phase and the global period of Experiment 2. Some authors have stated that acid oils present a lower nutritive value than native oils due to their main lipid molecular structure being FFA, negatively affecting FA absorption and energy utilization [6,24].

Regarding abdominal fat deposition, results indicated that the different added fats included had no influence. It was demonstrated by Ferrini et al. [25] that animals fed a diet high in SFA content (PUFA:SFA = 0.25) presented a higher AFP deposition than animals fed diets rich in PUFA (PUFA:SFA = 6.72). The lack of differences observed in fat deposition in the present studies could be related to the slight changes in saturation degree between treatments (grower–finisher S3 and L3 PUFA:SFA = 1.95 and 1.70, respectively).

### 4.3. Digestibility Balances

Results extracted from Experiment 1 showed that, in terms of FA and energy utilization, the substitution of S by L at any level in starter diets is not recommended. However, the results in adult broilers suggest that L can partially replace S up to 2%. In accordance with our results, Huang et al. [4] observed, in young broiler chickens, that the partial (1%) and total replacement (2%) of soybean oil by soybean lecithin reduced the feed AME content. In the case of adult broilers, they reported that the partial (0.5% and 1%) and total (2%), replacement of soybean oil by soybean lecithin did not affect the feed AME value or ether extract utilization. Tancharoenrat et al. [26] indicated that young chicks present a limited capacity to digest and absorb fats; however, this capacity is improved from two weeks of life. Our results are consistent with this fact due to the fact that grower–finisher broilers showed a better utilization of L than starter broilers.

In Experiment 2, the comparison between S3 and AO3 demonstrated the lowering effect of the high FFA content on the FA digestibility and the feed AME, as other authors have previously stated [27,28]. It has been established that the presence of monoacylglycerols is essential for a correct solubilization of the products derived from the lipolysis into mixed-micelles [24]. In addition, Sklan [24] also suggested a direct relationship between monoacylglycerol presence in the duodenum and bile secretion, justifying the lower FA absorption rate of acid oils in comparison to crude oil. These facts were confirmed by Rodriguez-Sanchez et al. [9], who observed that a high presence of FFA was related to an insufficient solubilization and absorption of lipolysis products, and, in particular, this fact was more pronounced with unsaturated diets than saturated ones. The blending of AO and L in starter diets did not modify the FA digestibility except for linolenic acid, which was enhanced by the L inclusion. However, in grower–finisher diets, the blending of 1% of AO and 2% of L resulted in the best option in terms of FA utilization. Some authors have suggested that soybean lecithin, as an emulsifier, may enhance lipid absorption—in particular, SFA and long-chain FA—by facilitating FA incorporation inside the micelles [5,6]. However, in accordance with Soares and Lopez-Bote results [3], no improvement of the SFA digestibility related to L inclusion was demonstrated in the present experiments. This lack of effect could be related to the highly unsaturated degree of the experimental diets used in the present study. On the other hand, in the grower–finisher phase, the AO1–L2 treatment resulted in the best option, thanks to an improvement in linolenic acid, along with a tendency for a growth of the PUFA digestibility (*p* = 0.071), which suggests an emulsifying effect. It is well known that blending fats with a complementary FA profile and different lipid molecular structures (triacylglycerols, FFA and PL) produces positive interactions in terms of the AME content and the FA digestibility [2,27,28]. The synergic effect observed between 1% of AO and 2% of L can be explained because it might have been an adequate proportion of PL capable of better solubilizing FFA in the mixed micelle, facilitating its absorption. On the other hand, it is important to comment on the grower–finisher results shown in the AO2–L1 treatment, which was also a blending treatment but showed the lowest feed AME value and the lowest TFA digestibility. Results may suggest that replacing an acidic oil by a less energetic oil with a high acidity, such as L (Table 2), caused an elevated proportion of the FFA:PL, thus leading to an insufficient presence of PL capable of solubilizing the FFA into the mixed micelles. As a consequence, a chemical characterization of the different fats and oils used as energy sources can provide important information about the possible interactions between different lipid molecular structures, as Roll et al. [28] have previously stated.

### 4.4. Fatty acid Composition of Abdominal Fat Adipose Tissue

The FA profile of the AFP reflected the FA profile of the diets, in accordance with most of the published data [25,29]. Though some authors have reported that the presence of different dietary lipid molecular structures, such as randomized FA, influences the FA profile of the AFP [29,30], our results demonstrate that the saturation degree of the AFP is more influenced by the dietary saturation degree rather than by the lipid molecular structures (triacylglycerols, FFA and PL) present in the feed. 

## 5. Conclusions

In summary, the inclusion of soybean lecithin high in FFA is suitable in grower–finisher diets as a partial replacer of soybean oil up to 2% without impairing performance, FA and energy utilization. Regarding to the use of a combination of co-products as an energy source, the best strategy in grower–finisher diets is a blend of 2% of high FFA soybean lecithin and 1% of monounsaturated vegetable acid oil; this is due to synergistic interactions on FA and energy utilization. Finally, the FA profile of the diets has a stronger impact on the FA profile of the AFP rather than the different lipid molecular structures.

## Figures and Tables

**Table 1:** Ingredient composition of the starter and grower–finisher broiler chicken diets on an as-fed basis (Experiments 1 and 2).

Ingredients (%)	Experiment 1		Experiment 2	
	Starter Diet (0–21 d)	Grower–Finisher Diet (22–38 d)	Starter Diet (0–21 d)	Grower–Finisher Diet (22–38 d)
Wheat	36.55	46.84	36.64	45.92
Soybean meal 47%	29.43	21.09	30.46	24.25
Corn	9.71	-	9.71	-
Barley	9.71	15.58	8.33	15.76
Extruded full-fat soybean	4.76	-	4.73	-
Added fats ^1^	3.00	3.00	3.00	3.00
Rapeseed meal 00	-	3.42	-	3.41
Sunflower meal 28%	-	2.44	-	-
Sepiolite	1.93	1.90	2.03	2.03
Palm oil	-	1.50	-	1.51
Calcium carbonate	1.19	1.08	1.16	1.00
Monocalcium phosphate	0.97	0.57	0.93	0.48
Trace minerals/vitamin premix ^2^	1.15	1.01	1.44	1.17
Titanium dioxide	0.50	0.50	0.50	0.50
Salt	0.30	0.23	0.30	0.23
L-lysine	0.30	0.35	0.28	0.28
DL-methionine	0.28	0.21	0.28	0.22
L-threonine	0.08	0.09	0.07	0.07
Sodic bicarbonate	0.07	0.12	0.07	0.11
Clorure choline 75%	0.07	0.07	0.07	0.06

^1^ Soybean oil and soybean lecithin high in free fatty acids and monounsaturated acid oil in different blending proportions. ^2^ Provides per kg feed: Vitamin A (from retinol), 13,500 IU; vitamin D3 (from cholecalciferol), 4800 IU; vitamin E (from alfa-tocopherol), 49.5 IU; vitamin B1, 3 mg; vitamin B2, 9 mg; vitamin B6, 4.5 mg; vitamin B12, 16.5 µg; vitamin K3, 3 mg; calcium pantothenate, 16.5 mg; nicotinic acid, 51 mg; folic acid, 1.8 mg; biotin, 30 µg; Fe (from FeSO_4_·7 H_2_O), 54 mg; I [from Ca(I_2_O_3_)_2_], 1.2 mg; Co (from 2 CoCO_3_·3 Co(OH)_2_·H_2_O), 0.6 mg; Cu (from CuSO_4_·5 H_2_O), 12 mg; Mn (from MnO), 90 mg; Zn (from ZnO), 66 mg; Se (from Na_2_SeO_3_), 0.18 mg; Mo [from (NH_4_)_6_Mo_7_O_24_], 1.2 mg; organic acids (starter diets at 4 g/kg; grower–finisher diets at 3 g/kg); β-glucanase 350 IU; xylanase 1125 IU.

**Table 2:** Chemical analysis of the experimental added oils.

Experimental Fats ^1^					
Item	Experiment 1		Experiment 2		
	S	L	S	AO	L
Fatty Acid Profile (%) ^2^					
SFA	16.5	20.4	16.0	15.1	21.9
C16:0	11.7	15.7	10.6	9.97	16.1
C18:0	3.55	4.68	4.26	3.84	5.86
MUFA	24.3	19.4	23.5	54.2	19.6
C18:1 ω-9	22.3	19.4	21.8	51.3	19.6
PUFA	59.2	60.2	60.5	30.7	58.5
C18:2 ω-6	53.4	54.2	52.8	29.2	52.6
C18:3 ω-3	5.76	6.09	7.67	1.55	5.90
Minor FA	3.32	N.D.	2.85	4.21	N.D.
UFA:SFA	5.06	3.90	5.25	5.62	3.57
PUFA:SFA	3.59	2.95	3.78	2.03	2.67
Acidity (%) ^2^					
FFA	2.41	22.6	1.49	52.9	25.5
Phospholipids (%) ^2^					
AI	N.D.	48.7	N.D.	N.D.	46.8
Total PL	N.D.	24.6	N.D.	N.D.	27.8
PC	N.D.	9.42	N.D.	N.D.	9.96
PI	N.D.	5.80	N.D.	N.D.	7.38
PE	N.D.	4.62	N.D.	N.D.	5.56
AP	N.D.	2.11	N.D.	N.D.	3.58
LPC	N.D.	2.68	N.D.	N.D.	1.31
Energy Content (MJ/kg)					
GE	39.3	34.0	40.3	39.5	34.7

SFA: Saturated fatty acid; MUFA: Monounsaturated fatty acid; PUFA: Polyunsaturated fatty acid; UFA:SFA: Unsaturated-to-saturated fatty acid ratio; PUFA:SFA: Polyunsaturated-to-saturated fatty acid ratio; FFA: Free fatty acid; AI: Acetone insoluble matter; Total PL: Total phospholipids; PC: Phosphatidylcholine; PI: Phosphatidylinositol; PE: Phosphatidylethanolamine; AP: Phosphatidic acid; LPC: Lysophosphatidylcholine; GE: Gross energy; N.D.: Not determined. ^1^ S: Soybean oil; L: Soybean lecithin high in free fatty acids; AO: Monounsaturated acid oil. ^2^ Percentage of total product.

**Table 3:** Analyzed gross energy, macronutrient content and fatty acid composition of starter and grower–finisher broiler chicken diets (Experiment 1).

Dietary Treatments ^1^								
Item *^2^*	Starter (0–21 d)				Grower–Finisher (22–38 d)			
	S3	S2–L1	S1–L2	L3	S3	S2–L1	S1–L2	L3
Macronutrient Content (%)								
Dry Matter	91.7	91.4	91.2	91.3	90.6	90.2	90.7	90.3
Crude Protein	23.7	23.1	22.7	23.2	21.5	20.8	21.4	20.7
Crude Fat	5.37	5.33	4.91	5.38	6.24	6.03	5.82	5.75
Crude Fiber	3.94	4.20	3.50	3.95	4.88	3.75	4.86	3.80
Ash	8.54	8.86	8.51	8.97	8.71	8.53	8.22	8.87
Fatty Acid Profile (%)								
SFA	18.2	18.7	19.1	19.9	25.0	25.9	27.0	28.0
C16:0	13.9	14.4	15.0	15.6	20.6	21.5	22.5	23.2
C18:0	3.55	3.58	3.70	3.88	3.44	3.51	3.55	3.80
MUFA	21.1	20.4	19.6	18.7	26.3	25.8	25.4	24.4
C18:1 ω-9	19.4	18.8	18.1	17.2	24.4	24.0	23.7	22.9
PUFA	60.7	60.9	61.3	61.4	48.7	48.3	47.6	47.6
C18:2 ω-6	54.6	54.7	54.7	54.7	44.2	43.8	43.1	43.0
C18:3 ω-3	6.16	6.18	6.23	6.39	4.80	4.68	4.64	4.75
Minor fatty acids	2.39	2.34	2.27	2.23	2.56	2.51	2.51	2.35
UFA:SFA	4.49	4.35	4.24	4.03	3.00	2.86	2.70	2.55
PUFA:SFA	3.34	3.26	3.21	3.09	1.95	1.86	1.76	1.70
Energy Content (MJ/kg)								
GE	17.4	17.3	17.1	17.1	17.5	17.4	17.4	17.2

SFA: Saturated fatty acids; MUFA: Monounsaturated fatty acids; PUFA: Polyunsaturated fatty acids; UFA:SFA: Unsaturated-to-saturated fatty acid ratio; PUFA:SFA: Polyunsaturated-to-saturated fatty acid ratio; GE: Gross energy. ^1^ S3: Soybean oil (S) at 3.00%; S2–L1: S at 2.00% and soybean lecithin high in free fatty acids (L) at 1.00%; S1–L2: S at 1.00% and L at 2.00%; L3: L at 3.00%. ^2^ Samples were analyzed twice.

**Table 4:** Analyzed gross energy, macronutrient content, and fatty acid composition of starter and grower–finisher broiler chicken diets (Experiment 2).

Dietary Treatments ^1^										
Item ^2^	Starter (0–21 d)					Grower–Finisher (22–38 d)				
	S3	AO3	AO2–L1	AO1–L2	L3	S3	AO3	AO2–L1	AO1–L2	L3
Macronutrient Content (%)										
Dry Matter	91.8	91.8	91.4	92.1	91.0	90.9	90.9	90.9	90.7	91.0
Crude Protein	22.3	22.2	23.1	22.5	22.5	22.1	21.3	20.6	20.6	20.8
Crude Fat	5.46	5.33	5.58	5.30	5.10	6.60	6.60	6.37	6.15	6.23
Crude Fiber	4.38	3.84	4.04	3.98	4.13	4.62	4.17	3.81	4.02	4.20
Ash	8.50	8.36	8.73	8.53	8.65	9.86	9.21	10.2	10.5	9.53
Fatty Acid Profile (%)										
SFA	17.2	17.9	18.2	18.8	19.6	23.6	24.7	25.4	25.9	26.5
C16:0	13.5	14.3	14.6	15.1	15.7	19.4	20.5	21.1	21.6	22.2
C18:0	3.70	3.66	3.63	3.68	3.87	3.96	3.95	3.98	3.99	3.99
MUFA	22.1	35.0	30.5	25.5	19.9	27.1	40.1	35.1	30.5	25.4
C18:1 ω-9	20.7	33.5	29.1	24.1	18.7	25.6	38.3	33.4	29.0	24.0
PUFA	60.7	47.1	51.3	55.7	60.5	49.3	35.2	39.5	43.6	48.1
C18:2 ω-6	54.1	43.4	46.8	50.6	54.6	43.7	32.5	36.0	39.6	43.3
C18:3 ω-3	6.61	3.74	4.45	5.14	5.90	5.56	2.73	3.46	4.07	4.81
Minor fatty acids	1.39	1.40	1.42	1.38	1.23	1.78	2.02	2.06	1.74	1.70
UFA:SFA	4.81	4.59	4.49	4.32	4.10	3.24	3.05	2.94	2.86	2.77
PUFA:SFA	3.53	2.63	2.82	2.96	3.09	2.09	1.43	1.56	1.68	1.82
Energy Content (MJ/kg)										
GE	17.3	17.3	17.1	17.1	16.9	17.6	17.6	17.5	17.5	17.4

SFA: Saturated fatty acids; MUFA: Monounsaturated fatty acids; PUFA: Polyunsaturated fatty acids; UFA:SFA: Unsaturated-to-saturated fatty acid ratio; PUFA:SFA: Polyunsaturated-to-saturated fatty acid ratio; GE: Gross energy. ^1^ S3: Soybean (S) oil at 3.00%; AO3: Acid oil (AO) at 3.00%; AO2–L1: AO at 2.00% and soybean lecithin high in free fatty acids (L) at 1.00%; AO1–L2: AO at 1.00% and L at 2.00%; L3: L at 3.00%. ^2^ Samples were analyzed twice.

**Table 5:** Growth performance and abdominal fat pad deposition of broiler chickens according to different dietary added fats (Experiment 1).

Dietary Treatments ^1^						
Item	S3	S2–L1	S1–L2	L3	RSE	*p*-Value
From 0 to 21 d						
BW at 0 d (g)	43.0	42.9	42.9	43.1	2.64	0.996
BW at 21 d (g)	825	816	836	825	85.6	0.891
ADFI (g/bird/d)	54.9	55.7	52.5	54.3	3.03	0.338
ADG (g/bird/d)	37.1	37.7	38.3	36.6	2.19	0.618
FCR (g/g)	1.45	1.41	1.40	1.44	0.039	0.170
From 22 to 38 d						
BW at 38 d (g)	2408	2461	2500	2428	186.8	0.509
ADFI (g/bird/d)	167.1	172.0	170.6	171.1	10.14	0.855
ADG (g/bird/d)	93.8	94.5	95.6	91.9	5.75	0.724
FCR (g/g)	1.78	1.79	1.82	1.86	0.075	0.287
From 0 to 38 d						
ADFI (g/bird/d)	105.1	107.8	105.3	105.9	6.00	0.885
ADG (g/bird/d)	62.3	61.8	63.9	62.2	3.70	0.755
FCR (g/g)	1.69	1.71	1.68	1.70	0.054	0.780
Carcass weight (g)	2147	2224	2241	2173	109.1	0.463
Abdominal Fat Depot						
g	40.01	35.86	33.78	39.45	3.835	0.062
(%)	1.93	1.61	1.64	1.82	0.251	0.134

BW: Body weight; ADFI: Average daily feed intake; ADG: Average daily gain; FCR: Feed conversion ratio; RSE: Residual standard error. ^1^ S3: Soybean oil (S) at 3.00%; S2–L1: S at 2.00% and soybean lecithin high in free fatty acids (L) at 1.00%; S1–L2: S at 1.00% and L at 2.00%; L3: L at 3.00%.

**Table 6:** Growth performance and abdominal fat pad deposition of broiler chickens according to different dietary added fats (Experiment 2).

Dietary Treatments ^1^							
Item	S3 ^2^	AO3	AO2–L1	AO1–L2	L3	RSE	*p*-Value
From 0 to 21 d							
BW at 0 d (g)	45.1	45.2	45.1	45.1	45.1	2.44	0.999
BW at 21 d (g)	876	878	870	864	876	89.6	0.943
ADFI (g/bird/d)	56.2	57.1	57.7	55.6	57.6	2.35	0.400
ADG (g/bird/d)	39.6	39.7	39.3	39.0	40.7	2.06	0.561
FCR (g/g)	1.40	1.44	1.47	1.41	1.45	0.052	0.943
From 22 to 38 d							
BW at 38 d (g)	2469	2395	2430	2418	2469	186.1	0.927
ADFI (g/bird/d)	163.5	160.9	165.4	159.8	164.7	9.90	0.706
ADG (g/bird/d)	91.7	87.8	90.3	89.8	90.0	6.36	0.897
FCR (g/g)	1.78 ^x^	1.86 ^y^	1.83	1.80	1.81	0.033	0.171
From 0 to 38 d							
ADFI (g/bird/d)	104.2	103.5	105.9	102.2	104.6	5.41	0.679
ADG (g/bird/d)	62.7	61.2	62.1	62.5	61.8	4.09	0.954
FCR (g/g)	1.66 ^x^	1.71 ^y^	1.71	1.66	1.67	0.032	0.055
Carcass weight (g)	2229	2183	2193	2172	2193	141.3	0.999
Abdominal Fat Depot							
g	43.86	40.61	45.04	39.34	45.31	5.528	0.185
(%)	1.97	1.88	2.05	1.79	2.06	0.175	0.064

^1^ S3: Soybean oil (S) at 3.00%; AO3: Acid oil (AO) at 3.00%; AO2–L1: AO at 2.00% and L at 1.00%; AO1–L2: AO at 1.00% and L at 2.00%. L3: L at 3.00%. ^2^ S3 was not included in the statistical analysis against diets containing co- and by-products. ^x,y^ ANOVA AO3 vs S3: Values within the same row with no common superscripts are significantly different, *p* ≤ 0.05. BW: Body weight; ADFI: Average daily feed intake; ADG: Average daily gain; FCR: Feed conversion ratio; RSE: Residual standard error.

**Table 7:** Feed apparent metabolizable energy value and fatty acid digestibility of starter and grower–finisher broiler chicken diets according to added fat sources (Experiment 1).

Dietary Treatments ^1^						
Item	S3	S2–L1	S1–L2	L3	RSE	*p*-Value
From 9 to 11 d						
AME (MJ/kg)	12.9 ^a^	11.6 ^b^	11.6 ^b^	11.4 ^b^	0.36	<0.001
Fatty Acid Digestibility (%)						
TFA	81.5 ^a^	77.5 ^a,b^	71.1 ^b^	70.9 ^b^	5.91	0.017
SFA	62.3	56.6	48.9	49.7	7.70	0.055
C16:0	69.5	65.0	58.4	60.4	6.83	0.098
C18:0	50.2	50.1	38.7	37.9	11.07	0.153
MUFA	79.2 ^a^	75.1 ^a,b^	67.9 ^b^	68.8 ^b^	6.50	0.026
C18:1 ω-9	80.3 ^a^	78.0 ^a^	69.8 ^b^	69.8 ^b^	3.98	<0.001
PUFA	88.0 ^a^	83.6 ^a,b^	75.6 ^b^	78.0 ^b^	5.45	0.004
C18:2 ω-6	87.7 ^a^	83.2 ^a,b^	74.9 ^b^	77.3 ^b^	5.60	0.003
C18:3 ω-3	90.6 ^a^	87.4 ^a,b^	80.6 ^b^	83.0 ^b^	4.31	0.003
From 36 to 37 d						
AME (MJ/kg)	13.0 ^a^	12.8 ^a^	12.9 ^a^	11.8 ^b^	0.39	<0.001
Fatty acid digestibility (%)						
TFA	85.0 ^a^	83.5 ^ab^	83.0 ^a,b^	79.0 ^b^	2.96	0.020
SFA	80.7	81.6	81.4	79.0	2.96	0.446
C16:0	82.3	83.3	83.3	81.0	2.84	0.480
C18:0	80.9	81.1	81.7	79.9	3.21	0.807
MUFA	84.8	83.8	81.8	78.7	4.25	0.141
C18:1 ω-9	88.5 ^a^	86.6 ^a^	87.9 ^a^	83.9 ^b^	1.46	<0.001
PUFA	85.3 ^a^	82.2 ^a,b^	84.7 ^a^	79.9 ^b^	2.29	0.003
C18:2 ω-6	85.1 ^a^	82.1 ^a,b^	84.5 ^a^	79.7 ^b^	2.33	0.003
C18:3 ω-3	86.4 ^a^	83.4 ^a,b^	85.7 ^a^	81.7 ^b^	2.00	0.003

^a^^–c^ Values within the same row with no common superscripts are significantly different, *p* ≤ 0.05. AME: Apparent metabolizable energy; TFA: Total fatty acid; SFA: Saturated fatty acid; MUFA: Monounsaturated fatty acid; PUFA: Polyunsaturated fatty acid; RSE: Residual standard error. **^1^** S3: Soybean oil (S) at 3.00%; S2–L1: S at 2.00% and soybean lecithin high in free fatty acids (L) at 1.00%; S1–L2: S at 1.00% and L at 2.00%; L3: L at 3.00%.

**Table 8:** Feed apparent metabolizable energy value and fatty acid digestibility of starter and grower–finisher broiler chicken diets according to added fat sources (Experiment 2).

Dietary Treatments ^1^							
Item	S3 ^2^	AO3	AO2–L1	AO1–L2	L3	RSE	*p*-Value
From 9 to 11 d							
AME (MJ/kg)	12.5 ^x^	12.0 ^y^	11.7	12.1	11.9	0.41	0.252
Fatty Acid Digestibility (%)							
TFA	79.6 ^x^	65.9 ^y^	66.1	71.4	70.1	7.06	0.478
SFA	68.1 ^x^	51.7 ^y^	53.4	59.9	60.6	9.62	0.344
C16:0	73.1 ^x^	61.5 ^y^	61.6	68.1	66.2	7.94	0.428
C18:0	70.3 ^x^	47.6 ^y^	58.8	62.6	62.3	12.69	0.186
MUFA	80.3 ^x^	70.5 ^y^	70.6	75.1	70.5	7.60	0.678
C18:1 ω-9	80.8	71.9	71.5	76.3	71.9	7.26	0.649
PUFA	82.6 ^x^	67.9 ^y^	68.0	75.4	73.8	5.67	0.097
C18:2 ω-6	82.1 ^x^	67.6 ^y^	67.7	73.3	73.3	6.13	0.234
C18:3 ω-3	86.7 ^x^	70.8 ^y,b^	71.3 ^b^	77.3 ^ab^	78.2 ^a^	4.12	0.011
From 36 to 37 d							
AME (MJ/kg)	12.7 ^x^	12.3 ^y,a^	11.7 ^b^	12.5 ^a^	12.4 ^a^	0.25	<0.001
Fatty Acid Digestibility (%)							
TFA	87.0 ^x^	84.3 ^y,a^	81.4 ^b^	84.6 ^a^	83.3 ^a,b^	1.73	0.022
SFA	83.3	81.1 ^a,b^	78.6 ^b^	82.8 ^a^	82.6 ^a^	1.80	0.002
C16:0	86.1	84.1 ^a^	81.4 ^b^	85.4 ^a^	85.0 ^a^	1.58	0.001
C18:0	88.1 ^x^	84.4 ^y,a,b^	84.0 ^b^	86.2 ^a^	86.2 ^a^	1.51	0.041
MUFA	88.2	88.6 ^a^	85.5 ^bc^	87.5 ^a,b^	84.7 ^c^	1.24	<0.001
C18:1 ω-9	90.1	90.0 ^a^	87.5 ^bc^	89.1 ^a,b^	87.1 ^c^	1.18	0.001
PUFA	87.9 ^x^	81.5 ^y^	79.3	82.9	82.9	2.52	0.071
C18:2 ω-6	87.5 ^x^	81.6 ^y^	79.2	82.8	82.6	2.52	0.080
C18:3 ω-3	90.5 ^x^	80.1 ^y,b^	80.1 ^b^	83.9 ^a,b^	84.9 ^a^	2.63	0.006

^1^ S3: Soybean oil at 3.00%; AO3: Acid oil (AO) at 3.00%; AO–L1: AO at 2.00% and soybean lecithin high in free fatty acids (L) at 1.00%; AO–L2: AO at 1.00% and L at 2.00%; L3: L at 3.00%. ^2^ S3 was not included in the statistical analysis against diets containing co-products. ^a^^–c^ Values within the same row with no common superscripts are significantly different, *p* ≤ 0.05; ^x,y^ ANOVA S3 vs. AO3: Values within the same row with no common superscripts are significantly different, *p* ≤ 0.05. AME: Apparent metabolizable energy; FA: Fatty acid; SFA: Saturated fatty acid; MUFA: Monounsaturated fatty acid; PUFA: Polyunsaturated fatty acid; RSE: Residual standard error.

**Table 9:** Fatty acid composition of abdominal fat pad of broiler chickens according to different fat sources ^1^ in diet (Experiments 1 and 2).

Item	Experiment ^1^						Experiment ^2^						
	Dietary Treatments				RSE	*p*-Value	Dietary Treatments					RSE	*p*-Value
	S3	S2–L1	S1–L2	L3			S3 ^2^	AO3	AO2–L1	AO1–L2	L3		
Fatty Acid Profile (%)													
SFA	29.8 ^b^	30.0 ^b^	30.3 ^b^	32.1 ^a^	1.06	0.005	29.2	29.8	30.8	30.3	31.1	1.14	0.287
C16:0	23.6 ^b^	24.0 ^b^	24.3 ^b^	25.7 ^a^	0.76	<0.001	23.1	23.6	24.2	23.9	24.5	0.98	0.463
C18:0	5.31	5.46	5.17	5.55	0.64	0.779	5.37	5.34	5.73	5.39	5.44	0.40	0.364
MUFA	44.9	46.4	46.4	46.9	2.81	0.661	46.8 ^y^	53.6 ^x,a^	50.8 ^a,b^	48.5 ^b^	46.7 ^b^	2.76	0.002
C18:1 ω-9	37.4	38.5	37.3	38.3	1.45	0.468	39.0 ^y^	45.4 ^x,a^	42.8 ^a,b^	40.6 ^bc^	38.6 ^c^	2.06	<0.001
PUFA	25.3	23.7	23.3	21.0	2.85	0.107	24.5 ^x^	16.6 ^y,b^	17.8 ^a,b^	21.4 ^a^	22.5 ^a^	2.75	0.004
C18:2 ω-6	22.4	20.9	19.7	18.5	2.40	0.069	21.3 ^x^	14.9 ^y,b^	16.0 ^a,b^	19.0 ^a^	19.9 ^a^	2.41	0.006
C18:3 ω-3	2.19	2.06	2.03	1.88	0.27	0.293	2.47 ^x^	1.06 ^y,c^	1.42 ^b,c^	1.81 ^a,b^	2.02 ^a^	0.26	<0.001
UFA:SFA	2.37 ^a^	2.34 ^a^	2.30 ^a^^,^^b^	2.11 ^b^	0.12	0.007	2.43	2.36	2.25	2.32	2.22	0.12	0.223
PUFA:SFA	0.85 ^a^	0.79 ^ab^	0.79 ^a^^,^^b^	0.65 ^b^	0.09	0.009	0.84 ^x^	0.55 ^y,b^	0.60 ^a,b^	0.74 ^a^	0.73 ^a^	0.10	0.014

^1^ S3: Soybean oil (S) at 3.00%; S2–L1: S at 2.00% and soybean lecithin high in free fatty acids (L) at 1.00%; S1–L2: S at 1.00% and L at 2.00%; AO3: Acid oil (AO) at 3.00%; AO2–L1: AO at 2.00% and L at 1.00%; AO1–L2: AO at 1.00% and L at 2.00%; L3: L at 3.00%. ^2^ S3 was not included in the statistical analysis against diets containing co-products. ^a–c^ Values within the same row with no common superscripts are significantly different, *p* ≤ 0.05. ^x,y^ ANOVA S3 vs. AO3: Values within the same row with no common superscripts are significantly different, *p* ≤ 0.05. SFA: Saturated fatty acid; MUFA: Monounsaturated fatty acid; PUFA: Polyunsaturated fatty acid; UFA:SFA: Unsaturated-to-saturated fatty acid ratio; PUFA:SFA: Polyunsaturated-to-saturated fatty acid ratio; RSE: Residual standard error.
